# Parkinson’s disease-associated alterations in DNA methylation and hydroxymethylation in human brain

**DOI:** 10.1038/s41531-025-01209-3

**Published:** 2025-12-22

**Authors:** Juliana I. Choza, Mahek Virani, Nathan C. Kuhn, Marie Adams, Joseph Kochmanski, Alison I. Bernstein

**Affiliations:** 1 Department of Pharmacology and Toxicology, Ernest Mario School of Pharmacy, Rutgers University, Piscataway, NJ USA; 2 Department of Translational Neuroscience, College of Human Medicine, Michigan State University, Grand Rapids, MI USA; 3 Genomics Core, Van Andel Research Institute, Grand Rapids, MI USA; 4 Environmental and Occupational Health Sciences Institute, Rutgers University, Piscataway, NJ USA

**Keywords:** Parkinson's disease, Epigenetics

## Abstract

Epigenetic mechanisms mediate interactions between aging, genetics, and environmental factors in sporadic Parkinson’s disease (PD). While multiple studies have explored DNA modifications in PD, few focus on 5-hydroxymethylcytosine (5hmc), which is important in the central nervous system and sensitive to environmental exposures. Existing studies have not differentiated between 5-methylcytosine (5mc) and 5hmc or have analyzed them separately. In this study, we modeled 5mc and 5hmc data simultaneously. We identified 108 cytosines with significant PD-associated shifts between these marks in an enriched neuronal population from PD postmortem parietal cortex, within 83 genes and 34 enhancers associated with 67 genes. These data potentially link epigenetic regulation of genes related to LRRK2 and endolysosomal sorting (*RAB32* and *AGAP1*), and genes involved in neuroinflammation, the inflammasome, and neurodevelopment with early changes in PD and suggest that there are significant shifts between 5mC and 5hmC associated with PD in genes not captured by standard methods.

## Introduction

An estimated 5–10% of Parkinson’s disease (PD) cases are familial and caused by monogenically inherited mutations, while the remaining ~90% of sporadic cases (sPD) are likely due to a complex interaction between age, genes, and environmental factors^1–3^. While the relative contribution of genetic and environmental risk factors in the etiology of sPD is debated, it is well documented that they play critical roles in the large majority of PD cases. Epigenetic mechanisms have emerged as critical mediators of the complex interactions between aging, genetics, and the environment because they are dynamic with age, sensitive to the environment, and regulate gene expression throughout the lifespan^4–6^. Evidence for the role of epigenetic regulation in PD has been building, particularly for DNA modifications^7–10^.

5-methylcytosine (5mC), the addition of a methyl group to the 5′-carbon of cytosine, is one of the most well-studied epigenetic marks. 5-hydroxymethylcytosine (5hmC) is formed via oxidation of 5mC by ten-eleven translocation (TET) enzymes and is a stable, independent epigenetic mark that has its highest levels in the brain, recruits a distinct set of DNA binding proteins from 5mC, differs in its genomic distribution in the brain compared 5mC, and is enriched in transcriptionally active gene bodies in the nervous system, suggesting a specific regulatory role for 5hmC in the brain^11^. Thus, 5hmC is now thought to be particularly important in gene regulation in the brain, particularly in the response to environmental exposures and neurotoxicants^12,13^. However, most studies of DNA modifications in PD brain have relied on bisulfite (BS) conversion, which cannot distinguish between 5mC and 5hmC^14,15^.

Recently, studies have begun to explore links between 5hmC and PD^16–18^. First, rare variants in *TET1* were associated with an increased risk of PD in a Chinese PD cohort^16^. Second, a targeted analysis of DNA modifications within known enhancers in human postmortem prefrontal cortex identified epigenetic disruption of an enhancer targeting the *TET2* gene in PD patients^18^. This study also performed hydroxymethylated DNA immunoprecipitation-sequencing (hMeDIP-Seq) in prefrontal cortex and found that PD-associated-hydroxymethylated peaks were enriched in gene bodies, promoters, and enhancers. Third, a small study in human postmortem substantia nigra (SN) used hMe-Seal, a selective chemical labeling method, and identified thousands of differentially hydroxymethylated regions in genes related to the central nervous system and neuronal differentiation, neurogenesis, and development and maintenance of neurites and axons, although the widespread neurodegeneration in the SN by the time of PD diagnosis complicates interpretation of these results^17^. Regardless, taken together, these initial studies support a role for 5hmC in regulation of expression of genes important for PD pathogenesis and indicate that additional research is warranted.

In our previous study, we performed a neuron-specific epigenome-wide association study (EWAS) with the Illumina EPIC BeadChip array paired with BS conversion using enriched neuronal nuclei from human postmortem parietal cortex obtained from the Banner Sun Health Research Institute Brain Bank^19^. We identified largely sex-specific PD-associated changes in DNA modification in 434 unique genes, including genes previously implicated in PD, including *PARK7* (DJ-1), *SLC17A6* (VGLUT2), *PTPRN2* (IA-2β), and *NR4A2* (NURR-1), as well as genes involved in developmental pathways, neurotransmitter packaging and release, and axon/neuron projection guidance. However, we could not differentiate between 5mC and 5hmC because we used BS conversion.

Here, we report the results of an EWAS of 5hmC and 5mC in enriched neurons from PD brain using our recently proposed method for reconciling base-pair resolution 5mC and 5hmC data^20^. To our knowledge, this is the largest epigenome-wide analysis of 5hmC to date in neurons enriched from PD post-mortem brain. We utilized additional DNA isolated from the same samples and performed oxidative BS (oxBS) conversion paired with the Illumina EPIC BeadChip array to specifically measure 5mC^21,22^. oxBS adds an oxidation step with potassium perruthenate (KRuO_4_) that specifically oxidizes 5hmC, forming 5-formylcytosine, prior to BS conversion. BS then deaminates the remaining 5mC only, but not 5-formylcytosine, such that C and 5hmC are read as thymine, while only the 5mC is read as cytosine, providing a readout of “true” methylation. Subsequent comparison of BS and oxBS results allows estimation of 5hmC and to generate specific β_mC_ and β_hmC_.

**Fig. 1:**
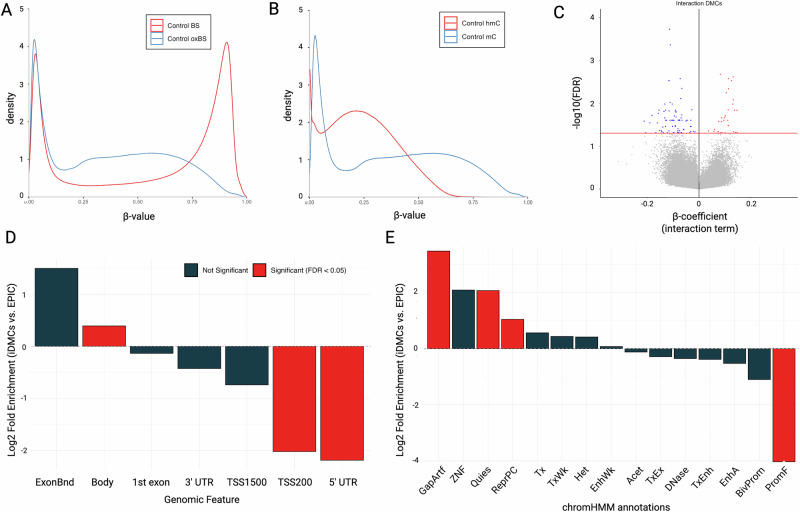
Identification of PD-associated iDMCs. Density plots of (**A**) raw BS and oxBS *β*-values and (**B**) MLE-corrected β_mC_ and β_hmC_ (**B**). **C** Volcano plot of iDMCs derived from MLE-corrected *β* values input into differential modification analysis (FDR < 0.05). Red indicates an increased interaction term; blue indicates a decreased interaction term. **D**, **E** Fold enrichment \ histograms showing significantly enriched annotations of significant iDMCs for (**D**) intragenic annotations and (E) universal chromatin state annotations.

**Fig. 2:**
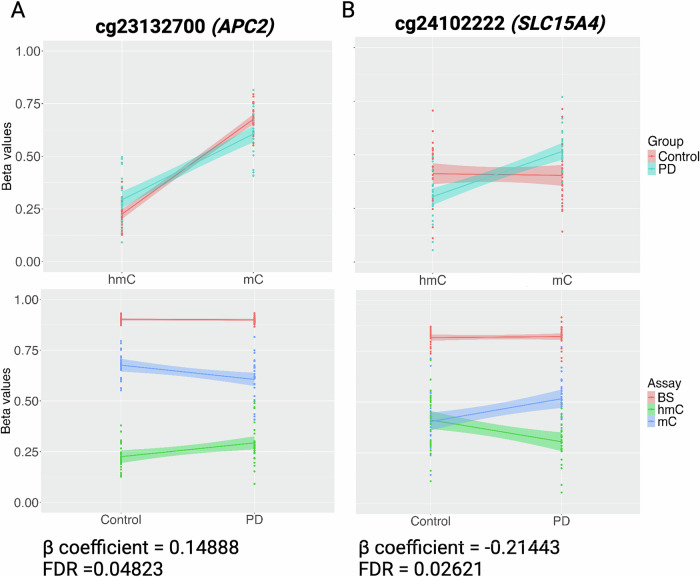
β-values of selected PD-associated DMCs. MLE-corrected βmC and βhmC values graphed by modification (top) and raw BS β values with MLE-corrected βmC and βhmC values graphed by disease status (bottom) for (**A**) the iDMC with the most positive interaction term and (**B**) the iDMC with the most negative interaction term (**B**). The corresponding β coefficient/interaction term and FDR value are indicated for each iDMC.

To identify differentially methylated and hydroxymethylated sites, most existing studies have either examined the distribution of 5hmC across the genome alone or analyzed 5mC and 5hmC separately^23–30^. However, these methods do not take into account the interdependence of 5mC and 5hmC *β* values or the differential distributions of *β*_*mC*_ *and β*_*hmC*_ (Fig. 1A, B). As a result, they fail to capture a complete picture of epigenetic regulation. This is most clearly seen when the combined proportion of 5mC and 5hmC (BS-derived *β* value) is unchanged, but there are shifts between different marks (*β*_*mC*_ *and β*_*hmC*_ values derived from paired BS and oxBS) (Fig. 2). As such, we previously developed a statistical approach using a mixed effects modeling approach to simultaneously model paired 5-mC and 5-hmC data as repeated measures that can detect these dynamic shifts in DNA modification cycling^20^. This method considers 5mC and 5hmC as paired data since these marks are biologically and statistically dependent on each other and has been used for EWAS in human cohorts exposed to lead and PFAS (per- and polyfluoroalkyl substances)^31,32^. Specifically, this model identifies changes in the interaction (β coefficient) between *β*_*mC*_ *and β*_*hmC*_ values with identified sites designated interaction differentially modified cytosines (iDMC). In this modeling, a positive β coefficient indicates a relative decrease in 5mC and an increase in 5hmC. In contrast, a negative interaction term indicates a relative increase in 5mC and a decrease in 5hmC (Fig. 2).

## Results

### Identification of iDMCs

In samples from male subjects, we identified 108 iDMCs with significant shifts in the proportions of 5mC and 5hmC associated with PD in DNA isolated from an enriched neuronal population derived from parietal cortex (FDR < 0.05) (Fig. 1C; Supplementary File 4). The majority of iDMCs were found in gene bodies and are located mainly in transcriptionally active chromatin (Fig. 1D,E).

As shown in Fig. 1C, more iDMCs have a negative than a positive β coefficient (76 vs 32, respectively). In this output, a positive β coefficient indicates a relative decrease in 5mC and an increase in 5hmC, as illustrated by visualizing raw BS *β* values and MLE-estimated *β* values for the iDMC with the largest positive β coefficient (Fig. 2A). In contrast, a negative interaction term indicates a relative increase in 5mC and a decrease in 5hmC, as illustrated by iDMC with the smallest negative β coefficient (Fig. 2B). iDMCs were enriched within gene bodies and regions annotated as quiescent or repressed polycomb, which are both areas of repressed transcription (Fig. 1D,E).

Due to the low sample size of female subjects after QC, we performed a targeted analysis of the 108 iDMCs identified in males in these samples. Of those, 29 were significant in female subjects using a lenient cutoff (*p* < 0.05). Of these, more iDMCs have a positive β coefficient than a negative β coefficient (20 vs 9) (Supplementary File 5). Twenty of these have β coefficients with the opposite direction of change in male and female subjects.

### Annotation of iDMCs

Of the 108 iDMCs, 71 were found within genes and were annotated to 83 genes, (Supplementary File 5). Using GREAT, an additional 67 genes were identified as potential targets of iDMC-containing enhancer regions (Supplementary File 7). Of these, 16 were also identified as iDMC-containing genes. Together, 134 unique genes were identified (Supplementary File 8). The 29 iDMCs that were also identified in females were annotated to 21 genes (Supplementary File 5).

### Overlap with previous EWAS studies

**Fig. 3:**
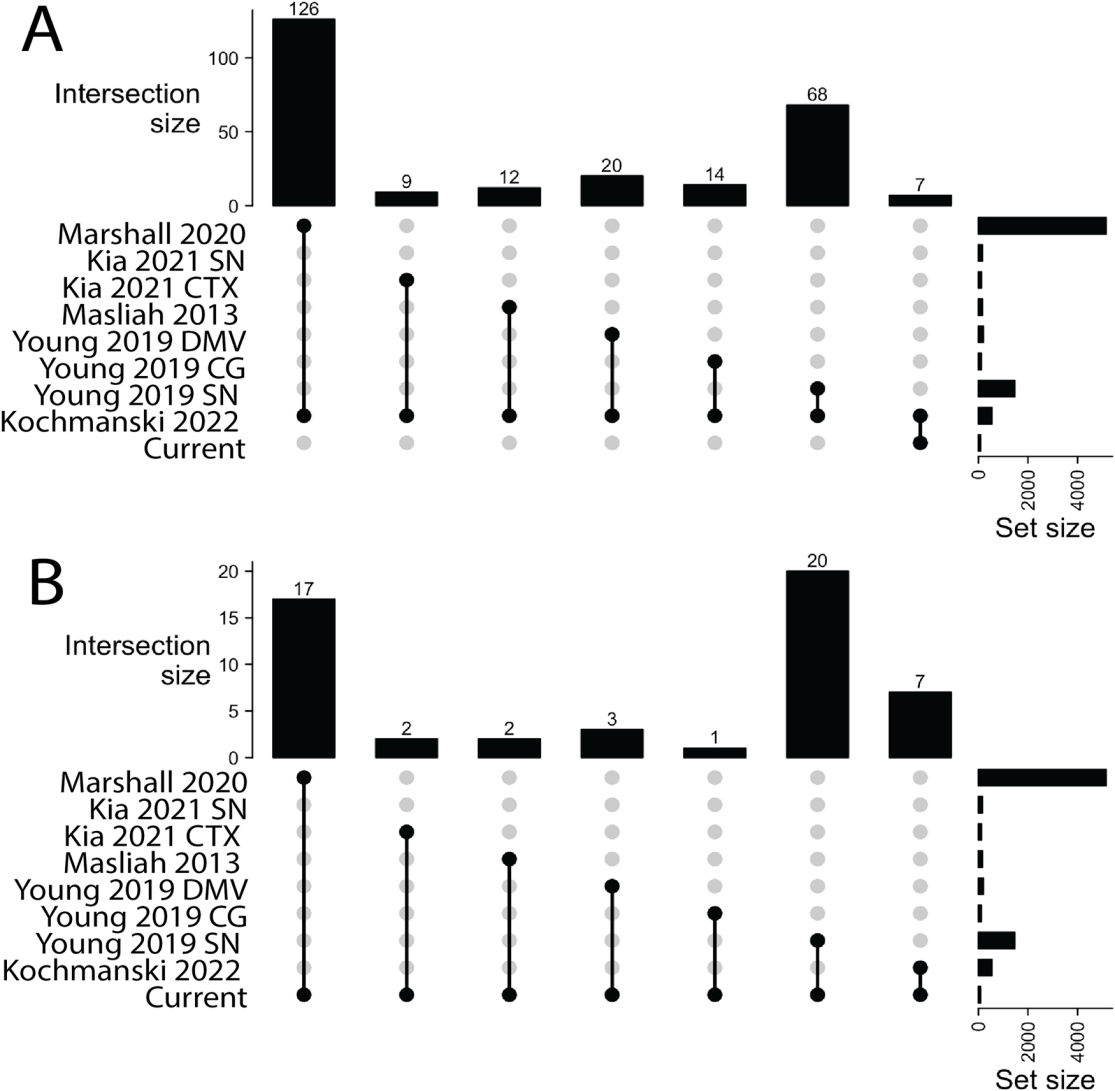
Overlap of BS-only study and current study with previous PD EWAS. UpSet plots show overlap of genes identified in (**A**) our previous BS-only analysis^19^ and (**B**) the current study with genes identified in previous PD EWAS studies.

Next, we compared these results with our BS-only results and other PD EWAS studies. First, data from our previous publication were reanalyzed. In the previous analysis, intergenic probes were removed prior to differential analysis, but here intergenic probes were included to allow for chromatin annotation of all regions^19^. While most of the DMCs, DMRs, and ~95% of genes identified were also identified in the original analysis, some new DMCs and DMRs were significant here, and some were no longer significant. The results of this updated analysis were used to compare interaction modeling to BS-only analysis. Annotated DMCs and DMRs from this BS-only analysis are included in Supplementary Files 9 and 10, respectively, and code is provided in Supplementary File 11. Of the 83 iDMC genes identified here, only 7 iDMC-containing genes were also identified by our BS-only analysis (*AGAP1*, *CACNA1H*, *COX19*, *LMF1*, *PMFBP1*, *RAB32*, and *TFAP2A*), albeit at different cytosines. Thus, these two methods identified largely unique sets of genes (Table 1, Fig. 3). As predicted, probes identified in this study, but not in the BS-only analysis, showed no change in the BS-derived *β* value, but changes in the proportion of each mark (*β*_*mC*_ *and β*_*hmC*_ values) (Table 1, Fig. 3).

**Table 1:** Summary of comparisons between the current study and previous PD EWAS in brain tissue

	5hmC	5mC and 5hmC (BS)							Paired BS/oxBS
	Marshall 2020	Kia 2021		Masliah 2013	Young 2019			Kochmanski 2022	Current study
**Control (M/F)**	23*	Not specified		6 (2/4)	41 (all male)			49 (29/20)	29 (all male)
**PD (M/F)**	20*	134*		5 (5/0)	38 (all male)			50 (33/17)	27 (all male)
**Region**	PFC	SN	CTX	FC	DMV	CG	SN	PC	PC
**PMI in hours (Control/PD)**	< = 48	Not specified		< = 8	<24			M: 3.28 ± 0.82/3.28 ± 0.86 F: 3.05 ± 0.97/ 3.19 ± 0.8	3.25 ± 0.81/3.27 ± 0.83
**Age (Control/PD)**	>50	Not specified		89 ± 4/80 ± 8	>60			M: 79.1 ± 9.1/79.2 ± 7.4 F: 82.2 ± 13.1/79.4 ± 5.5	79.3 ± 9.1/79.4 ± 7.1
**Disease stage**	Braak 3-6	Not specified		7−26 years (time since diagnosis)	Braak 3-5			Braak 2-3	Braak 2-3
**Sex as biological variable**	Sex included as covariate	Sex included as covariate		Not specified	All male			Sex stratified	All male
**Method**	hMe-DIP	BS-450K		BS-450K	BS-450K/EPIC			BS-EPIC	BS/oxBS-EPIC
**# genes**	5157	154	125	155	203	119	1466	547	83
**Overlap with BS**	126	5	9	12	20	14	68	-	7
**Overlap with interaction**	17	0	2	2	3	1	20	7	-

*Sex not specified, *SN* substantia nigra, *PFC* prefrontal cortex, *CTX* cortex, *FC* frontal cortex, *DMV* dorsal motor nucleus of the vagus, *CG* cingulate gyrus, *PC* parietal cortex.

Next, we compared the list of genes in this study to recent brain-specific EWAS studies for PD, including ours, for which data were provided^18,33–35^. When comparing the current study with 4 recent studies and our BS-only analysis, the most frequently identified genes across studies were *AGAP1, C10orf71, CACNA1H*, and *RAB32* (Table 1, Fig. 3, Supplementary File 12). Of the 4 recent studies, one specifically measured 5hmC by hMeDIP-seq; the others used BS conversion and did not differentiate between 5mC and 5hmC. Finally, 7 genes (*AGAP1, APC2, GNAS, ELANE, POLR2E, ZNF341*, and *WWOX*) were also identified in our two-hit mouse model of increased PD susceptibility^36,37^.

### Gene ontology enrichment analysis and protein-protein interaction networks

**Fig. 4:**
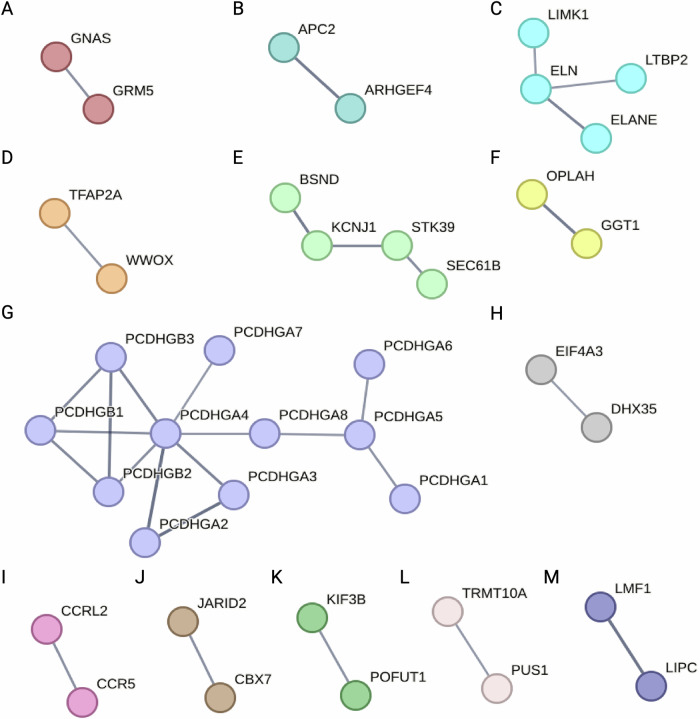
Protein interaction network for iDMC-containing genes and enhancer region target genes. String protein interaction network for 134 unique genes (confidence > = 0.7; > = 2 genes per subnetwork. All but 2 genes mapped to the STRING database (MIR7152, LOC102724297). Disconnected nodes in the network are hidden. PPI enrichment value = 2.95 × 10^−7^. Full network data and options can be viewed at: https://tinyurl.com/2w83atsn.

To explore known functions of these genes, we performed gene ontology enrichment analysis and generated protein-protein interaction networks. By gene ontology enrichment analysis for biological process, 36 genes were enriched in 16 GO terms within 8 GO term groups, including terms related to multiple cellular development pathways and chemokine signaling (Table 2). Enrichment analysis for cellular compartment identified 3 enriched GO Terms: catenin complex, catalytic step 2 spliceosome, and anchored compartment of plasma membrane (Table 3). Protein-protein interaction (PPI) networks were generated in STRING and are shown in Fig. 4^38^.

**Table 2:** Enriched GO Terms and GO Term Groups of identified genes based on the GO Biological Process term

GO Groups	GO Terms	Associated Genes	Group p-value (Bonferroni)
Group0	chemokine-mediated signaling pathway	CCR5, CCRL2, STK39, TFF2	0.016
Group1	calcium ion import	CACNA1H, PDGFB, TRPV3	0.021
Group2	neuromuscular process controlling balance	CDH23, GAA, JPH3	0.021
Group3	cell-cell adhesion via plasma-membrane adhesion molecules, homophilic cell adhesion via plasma membrane adhesion molecules	CDH23, FAT3, PCDHGA1, PCDHGA2, PCDHGA3, PCDHGA4, PCDHGA5, PCDHGA6, PCDHGA7, PCDHGA8, PCDHGB1, PCDHGB2, PCDHGB3, PCDHGB4	6.3 ×10^-8^
Group4	endoderm formation, endodermal cell differentiation	COL5A1, HSBP1, LAMA3	0.017
Group5	phosphatidic acid metabolic process, phosphatidic acid biosynthetic process	AGPAT4, LIPC, PLD1	0.014
Group6	autonomic nervous system development, cranial nerve development	GBX2, PHOX2A, TFAP2A, DRGX	0.016
Group7	cellular response to nutrient, response to vitamin D, cellular response to vitamin, vitamin D receptor signaling pathway, cellular response to vitamin D	KANK2, PIM1, RXRA	0.009

Results of ClueGO gene ontology enrichment analysis and significant grouped GO terms are shown (Bonferroni corrected *p*-value < 0.05). GO terms are grouped when they share >50% of their genes. “Associated Genes” shows all genes that map to each GO Term Group.

**Table 3:** Enriched GO Terms and GO Term Groups of identified genes based on the GO Cellular Component term

GO Groups	GO Terms	Associated Genes	Group *p*-value (Bonferroni)
Group0	catenin complex	APC2, CDH23, PCDHGB4	0.006
Group1	anchored component of plasma membrane	EEPD1, RTN4RL1, TEX101	0.010
Group2	catalytic step 2 spliceosome	DDX23, DHX35, EIF4A3, RBM44	0.007

Results of ClueGO gene ontology enrichment analysis and significant group GO terms are shown (Bonferroni corrected *p*-value < 0.05). GO terms are grouped when they share >50% of their genes. “Associated Genes” shows all genes that map to each GO Term Group.

### Endolysosomal genes

Two of the genes most frequently identified across PD EWAS studies encode the endolysosomal proteins RAB32 and AGAP1 (Supplementary File 12). We identified an iDMC located in a promoter of *RAB32*, showing a relative increase in 5mC and a decrease in 5hmC (Table 4). Epigenetic regulation of this gene in PD has also been reported in other brain EWAS, in our previous BS-only study, and peripheral immune cells (Table 1, Supplementary File 12)^18,19,35,38^. Recently, *RAB32* was identified as a causative gene for autosomal dominant PD^39–43^.

**Table 4:** Selected iDMCs of interest

Probe	Chr	Start	End	β coefficient	FDR	Gene Name	Region	Annotations
cg03447424	2	235930850	235930852	0.112	0.025	*AGAP1*	Body	Alternate exon, Transcription 3ʹ
cg23132700	19	1458041	1458043	0.149	0.048	*APC2*	Exon Boundary, Body	Transcribed 3ʹ Enhancer
cg03526702	19	856144	856146	−0.159	0.017	*ELANE*	Body	Quiescent/Low
cg07284407	20	58854802	58854804	−0.061	0.041	*GNAS*	1st Exon, 3’UTR	Bivalent Promoter Putative ICR
cg04113075	6	146544350	146544352	−0.070	0.049	*RAB32*	Body	Promoter
cg17419299	13	48302813	48302815	0.061	0.041	*RB1*	TSS1500	Active enhancer
cg24102222	12	128795903	128795905	−0.214	0.026	*SLC15A4*	Body	Active transcription (CG) Transcribed 3ʹ Enhancer (SN)
cg18173450	16	78389271	78389273	-0.124	0.049	*WWOX*	Body	Quiescent/Low

iDMCs highlighted in the text are listed with β coefficient, FDR, RefSeq Gene Name, region annotation, and brain-specific ChromHMM annotations indicated. Hg38 coordinates are shown. Additional annotations are from brain-specific ChromHMM annotations corresponding to imputed HMM in CG (E069) and SN (E074) and a database of ICRs. When annotations differ between CG and SN, region is indicated.

We also identified an iDMC in *AGAP1* within an alternate exon which shows a relative increase in 5hmC and a decrease in 5mC (Table 4). Alternative splicing of this transcript produces at least three variants, and this exon is present in at least two of these. Differential modification of this gene was also reported in other brain EWAS and our previous BS-only study (Table 1, Fig. 3, Supplementary File 12)^18,19,44^. AGAP1 is an Arf GTPase activating protein (ArfGAP) that has previously been associated with neurodevelopmental disorders, possibly by affecting dendritic spine morphology^45^. It is a direct regulator of adapter-related protein complex 3 (AP3) trafficking proteins, and of cytoskeletal remodeling^46,47^.

### Genes in neuroinflammatory pathways

The iDMC with the largest negative interaction term annotated to *SLC15A4* and is located within the last intron and annotated as active transcription in CG and transcribed 3ʹ enhancer in SN (Table 4). This iDMC was also identified in female subjects, but with an opposite β coefficient (Table 5, Supplementary_File_5). SLC15A4 has known interactions with NLRP inflammasome proteins, and additional proteins involved in inflammasome activation and neuroinflammation pathways in this dataset: TNFSF11 (TNF superfamily member 11), NFKBID (NF-κB inhibitor delta), IL16 (interleukin 16), CCR5 (C-C chemokine receptor type 5), and CCRL2 (C-C chemokine receptor-like 2). *CCR5* and *CCRL2* are annotated to the GO term “chemokine-mediated signaling pathway” along with *STK39;* these genes appear in two PPI networks (Fig. 4E,I, Table 2).

**Table 5:** Selected iDMCs of interest in both male and female

Male				Female				
Gene Name	β	SE	FDR	β	SE	*p*-value	probe	Feature
**ELANE**	−0.16	0.03	0.017	0.25	0.04	1.94 × 10^-=7^	cg03526702	Body
**GNAS**	−0.07	0.01	0.041	0.05	0.02	0.01	cg07284407	1stExon;3’UTR
**AGPAT4**	−0.14	0.03	0.049	0.16	0.04	3.64 × 10^-4^	cg09221482	Body
**WWOX**	−0.12	0.03	0.049	−0.11	0.05	0.03	cg18173450	Body
**SLC15A4**	−0.21	0.04	0.026	0.28	0.07	2.6 × 10^-4^	cg24102222	Body

iDMCs highlighted in the text are listed with β coefficient and standard errort, FDR for male analysis, *p*-value for targeted female analysis, RefSeq Gene Name, and feature annotation.

### Genes in lipid biosynthesis and homeostasis pathways

We identified an iDMC within a transcribed enhancer in both male and female subjects with opposite β coefficients within *AGPAT4* (Table 5, Supplementary_File_5). AGPAT4 encodes a member of the 1-Acylglycerol-3-Phosphate O-Acyltransferase family of genes and catalyzes the second step of de novo phospholipid biosynthesis, converting lysophosphatidic acid to phosphatidic acid^48^. We also identified iDMCs in enhancers predicted to regulate additional genes encoding lipid biosynthetic enzymes, *LIPC* and *PLD1*, which encode hepatic lipase and phospholipase D1, respectively. These three genes are annotated to the GO terms phosphatidic acid metabolic process and phosphatidic acid biosynthetic process (Table 2). Additional members of the AGPAT family (AGPAT1, AGPAT6) have been identified in previous PD EWAS studies^19,35^.

### Imprinted genes

The iDMC-containing imprinted gene *GNAS* is a highly complex imprinted locus with multiple transcripts derived from alternate promoters and 5′ exons, as well as an antisense transcript expressed from the opposite strand that encodes multiple forms of the alpha subunit of the stimulatory G protein (G_α_s)^49^. G_α_s acts to couple G protein-coupled receptors for multiple neurotransmitters with their second messenger systems, and proper imprinting plays a critical role in development. The identified iDMC shows a relative increase in 5mC and decrease in 5hmC and is located within a bivalent promoter and putative ICR in an alternate 5′ exon (Table 4, Supplementary File 4)^44^. This iDMC was also identified in female subjects, but with an opposite β coefficient (Table 5, Supplementary_File_5). In the PPI network, this gene interacts with another iDMC-containing gene *GRM5* (metabotropic glutamate receptor 5, mGluR5), which has been well-studied in the context of PD and L-DOPA-induced dyskinesias, with inconsistent results in clinical trials targeting mGluR5 (Fig. 4A)^50^.

An iDMC annotated to the imprinted gene *RB1* (RB transcriptional corepressor 1) is located within an active enhancer just upstream of the transcription start site for *RB1* and within an intron of the long non-coding RNA, *RB1* divergent transcript, and shows a relative increase in 5hmC and decrease in 5mC. While RB1 is extremely well studied in the context of cancer, its role in the nervous system and PD has also been explored, where it is essential for the survival of post-mitotic neurons^51–53^.

### Genes identified in our model of increased PD susceptibility

Multiple genes identified in this study were also identified in our two-hit mouse model of increased PD susceptibility and form PPI networks with other genes in the current dataset (*AGAP1, APC2, GNAS, ELANE*, and *WWOX*) (Fig. 4A–D)^36,37^. As discussed above, *GNAS* is an imprinted gene, and it has also been found to be differentially modified in other exposure models^54^. *APC2* contains the iDMC with the largest positive interaction term (Fig. 2A). It encodes the APC2 (APC Regulator Of WNT Signaling Pathway 2), which forms a complex with ARGHEF4 (Rho Guanine Nucleotide Exchange Factor 4) involved in E-cadherin-mediated cell-cell adhesion and regulation of microtubule dynamics with potential functions in axon guidance and dendritic formation during neurodevelopment (Fig. 4B)^55,56^.

*ELANE* contains an iDMC within an exon annotated as quiescent/low (Table 4). This iDMC was also identified in female subjects, but with an opposite β coefficient (Table 5, Supplemental Table 5). This gene encodes neutrophil elastase, which has putative connections to ELN (elastin), LIMK1 (LIM domain kinase 1), and LTBP2 (Latent Transforming Growth Factor Beta Binding Protein 2) (Fig. 4C). Elastin is one of the main structural components of many tissues, including brain blood vessels, and collectively, these proteins function in creating and maintaining the extracellular matrix (ECM). Within the nervous system, the ECM plays important roles in synaptic plasticity, growth of dendritic spines, and stabilization of synaptic connectivity^57^. More specifically, recent evidence implicates the degradation of elastin in aging, neuroinflammation, and age-related vascular diseases, but the role of elastin in neurodegenerative disease remains poorly studied^58^.

*WWOX* contains an iDMC with an intron specific to one *WWOX* transcript variant annotated as quiescent/low (Table 4). This gene encodes the WW domain-containing oxidoreductase, which is known to regulate *TFAP2* (Transcription factor AP-2-alpha) (Fig. 4D). This iDMC was also identified in female subjects, with the same direction of change (Table 5, Supplemental Table 5). While initially identified as a tumor suppressor, *WWOX* plays a role in a wide range of pathways and processes, including neurodevelopment and possibly neurodegeneration^59^. In addition, *TFAP2* is annotated to GO terms related to nervous system development (Table 3).

## Discussion

Here, we performed an integrated genome-wide analysis of 5mC and 5hmC using our novel application of mixed effects modeling in enriched neuronal nuclei from PD post-mortem parietal cortex samples, which has been used in our lab and others^20,31,32^. Because this region develops pathology late in PD, it is expected to still have robust populations of neurons in the mid-stage subjects selected for this study (unlike the substantia nigra, where neuron loss occurs early in disease). Thus, use of this region provides an avenue to investigate pre-pathological changes in gene regulation. The identified PD-associated iDMCs were largely unique from DMCs identified in our previous BS-based EWAS: 7 genes were identified in both studies, 76 only in the paired analysis, and 540 genes only in the BS-only analysis (Supplementary Files 7,8,11)^19^. Collectively, these data suggest that there are significant PD-associated shifts between 5mC and 5hmC at iDMCs that are not captured by analyzing BS-based data alone (Fig. 1C)^19,20^. These data suggest that shifts in the balance between DNA modifications may play an important but unrecognized role in PD etiology in both known and novel PD-related genes. While there are many genes of interest to explore in this dataset, we highlight a selection of genes based on known functions, gene-ontology enrichment, and protein-protein interaction results. Overall, these results indicate that the inclusion of epigenetic data expands known networks of genes and proteins that may be dysregulated in PD and can identify pathways not previously studied in PD. Importantly, because this study was performed in post-mortem brain tissue, the results shed light on potential mechanisms but are unlikely to be informative for the development of biomarkers.

We identified multiple genes involved in endolysosomal trafficking and LRRK2-mediated pathways, which are important in PD pathogenesis^60,61^. LRRK2 is the most commonly mutated gene in familial PD, and common variants are associated with sporadic PD^62^. While LRRK2 and these genes are not shown in the stringent interaction networks generated by STRING in Fig. 4, if we allow for interacting proteins and lower the stringency, there are known and potential connections between LRRK2 and the following proteins in our dataset: AGAP1, RAB32, RAB41, RADIL, and RAPGEF1. Specifically, RAB32 is a small GTPase that interacts with other PD genes (*LRRK2*, *PINK1*, *VPS35*) that are critical mediators of the endolysosomal sorting pathways known to be involved in PD^60,63,64^. *AGAP1* was identified as a differentially expressed gene in peripheral blood samples of fast- and slow-progressing PD patients^65^. Of particular interest for sporadic PD, defects in AGAP1 function have been proposed to render cells vulnerable to second-hit cytotoxicity and may contribute mechanistically to gene-environment interactions, and LRRK2 can be activated by PD-related toxicants^62,66^. The identification of epigenetic regulation of LRRK2-interacting genes suggests that epigenetic regulation of PD risk genes and associated pathways may represent a mechanistic link between genetic and environmental risks for PD.

*SLC15A4* (solute carrier family 15 member 4) contains the iDMC with the largest negative interaction term; SLC15A4 is an amino acid transporter within the endolysosomal membrane involved in the positive regulation of pattern recognition pathways (Fig. 2B). While SLC15A4 has primarily been studied in peripheral immune cells, it is expressed in many types of neurons, as verified in the Allen Brain Cell Atlas^67^. In peripheral immune cells, it is required for trafficking and colocalization of nucleic acid–sensing Toll-like receptors to endolysosmes in conjunction with AP3, and it promotes both inflammasome activity and increased autophagy in response to infection^68,69^. Within the brain, the inflammasome is typically thought of in the context of glial cells, but it is also important in neurons, including midbrain neurons^70–73^. Epigenetic regulation of these pathways is intriguing, especially given recent interest in the inflammasome as a mediator of gene-environment interactions in PD and ongoing studies of inflammasome inhibitors for PD^74–77^.

Imprinted genes are highly sensitive to environmental perturbation because their epigenetic marks are not cleared during development and are known to be critical for growth, metabolism, and neuronal function^78^. Many imprinted genes show distinct patterns of imprinting and expression in the brain compared to other tissues. Environmental disruption of imprinting during development leads to long-term and persistent changes in gene expression in pathways important in the pathogenesis of neurological diseases, providing a potential mechanism by which environmental exposures can impact the risk of late-life disease^79^. The identified iDMC in *GNAS* is located within bivalent promoter and putative ICR (Table 4). As a result, disruption of imprinting of the *GNAS* locus could lead to changes in imprinting and cell- and tissue-specific transcript expression, affecting development and GPCR-mediated neurotransmitter signaling pathways. The iDMC within the *RB1* locus is not located within the ICR, but epigenetic regulation of the iDMC-containing promoter is known to regulate chromosomal looping and expression of RB1, and disruption of this looping can lead to decreased expression and tumorigenesis^44,80^. Thus, it is possible that epigenetic dysregulation in the brain of the chromosomal looping that regulates *RB1* expression could lead to cell loss via cell cycle reentry and senescence^52,81^.

The significant effects of sex on the epigenome are well established. While our ability to assess sex differences in this study was limited by low sample size in female subjects, a targeted analysis of the 108 iDMCs identified 29 (~25%) of these in female subjects, with 20 showing the opposite direction of change. These results are consistent with our previous study, which showed distinct sex-specific effects on the epigenome in PD, as well as studies of other neurodegenerative diseases^19,82–84^. Given the well-documented sex differences in both susceptibility to and progression of PD, these and previous results together suggest that epigenetic regulation may underlie these sex differences^82,83,85^.

While we assessed neuron-specific DNA modifications associated with PD in a region without widespread neuron loss prior to the onset of pathology within the parietal cortex in an attempt to capture early, pre-degenerative changes, post-mortem studies do not allow for longitudinal analysis of the progressive changes that lead to disease. To model this, we developed a two-hit mouse model of environmentally induced increased PD susceptibility in which developmental exposure to the organochlorine pesticide dieldrin leads to a male-specific exacerbation of neurotoxicity induced by synucleinopathy in the α-synuclein preformed fibril (α-syn PFF) model^37,84,86^. Most recently, we identified dieldrin-induced changes in DNA modifications from birth to 9 months of age in pathways related to early neurodevelopment, dopaminergic neuron differentiation, synaptogenesis, synaptic plasticity, and glial-neuron interactions, consistent with the hypothesis that increased susceptibility to late-onset neurological diseases has origins in development^36^. The genes and pathways shared between our model and human PD have known functions in neurodevelopment and the establishment and maintenance of synapse and neural circuits, providing insight into mechanisms that may set the stage for increased susceptibility to disease (Table 4, Fig. 4C).

While these changes were not assessed in the SN, the region most commonly studied in the context of PD, by using the parietal cortex, which does not have widespread degeneration at this stage of disease, we were able to assess neuron-specific DNA modifications associated with PD in a region without widespread neuron loss. Thus, overall, these data suggest that PD-associated alterations in the epigenetic regulation of these genes may alter gene expression, promoter usage, or isoform expression of these genes and may represent early pre-degenerative events that precede the onset of degeneration.

The Illumina EPIC array is commonly used in EWAS studies due to its cost, ease of analysis, and reproducibility. Use of this method allows for integration with existing and future studies using this commonly used Illumina BeadChip technology. However, there are specific limitations of this study due to the use of this platform. The EPIC array does not provide a true genome-wide analysis as it covers <5% of the cytosines in the human genome. Complete genome-wide analysis requires a whole-genome method, such as whole genome BS sequencing or enzymatic methyl-Seq. However, while costs have dropped dramatically, these methods remain cost-prohibitive for large cohorts, making it unfeasible for this project, which involved running 200 arrays. In addition, this data does not provide complete genomic coverage across the genome or the identified target genes because of the design of the EPIC array. This is an inherent limitation of this platform. As such, future work should include locus-specific targeted analysis to assess all cytosines at identified loci and genomic features. Finally, this array was not designed specifically for brain-specific and neurodegenerative disease studies, and some genomic regions of interest to the PD field may not be covered on the array.

An additional caveat of these findings is that this study does not address the biological significance of these epigenetic shifts. While shifts between 5mC and 5hmC may potentially impact the binding of proteins that regulate gene expression and/or other epigenetic marks, this study does not examine these functional impacts or identify the cell-type specificity of these changes. However, it does provide multiple avenues for further study of the impact of these changes on gene expression, alternate promoter usage, differential isoform expression, and neuronal function and susceptibility. In addition, emerging spatial and single-cell methods for examining DNA modifications can be paired with spatial and single-cell transcriptomics, to provide potential avenues for examining the cell-type specific functional impacts of these epigenetic shifts.

It is not surprising that the overlap between our data and these other EWAS was minimal (Table 1, Fig. 3, Supplementary File 12). There are major differences in existing PD EWAS related to sample size, sample selection, age, post-mortem interval, brain region assessed, disease stage, sex ratio, methods used to measure DNA modifications, statistical modeling, significance thresholds, and inconsistent reporting between studies (Table 1). For example, one study performed hMe-DIP in prefrontal cortex to assess genome-wide 5hmC, while three BS-based studies used either the EPIC array or the previous 450 K array in SN, frontal cortex, the dorsal motor nucleus of the vagus (DMV), and cingulate gyrus (CG)^18,33–35^. The methodological differences between these studies complicate the comparison. This is particularly true for oxBS and 5hmC analyses, as analysis parameters and cutoffs vary between labs. While multiple papers have recommended statistical cutoffs and best practices for EWAS studies based on BS data, little guidance exists for oxBS analysis^87–89^. Standardizing cutoffs across studies will be critical moving forward in this field, as this can profoundly affect the interpretation of results. For example, we selected FDR < 0.05 in this current study, but if we set stringency at 1%, there are 15 significant probes or at 10%, there are 309. Together, these issues underscore the need for rigorous, open, and reproducible methodology and analysis, as discussed in recent chapters from our group and others on rigor and reproducibility in EWAS^87,88^.

In addition, it is also important to note that this study included a high level of failed probes and failed samples unique to the oxBS reactions. In contrast, in the BS data, no samples were excluded due to high levels of failed probes, and far fewer probes failed. As noted in the methods, we started with 1 µg of input DNA for oxBS because when we used the recommended starting amount of 500 ng, all oxBS probes failed. Together, this suggests that oxBS is much harsher on the DNA that BS alone, requiring high input amounts that may limit the utility of this method as the field moves towards cell type-specific methods and lower input amounts. Because of these technical issues with the initial batch of oxBS reactions, BS and oxBS reactions were run on a separate batch of nuclei and DNA isolations. Ideally, these paired reactions would be run on the same batch of isolations. As a result, this batch effect is a technical variable we are unable to control for in this study. The inability to control for this cell type heterogeneity may contribute to some of the observed genomic inflation (Supplementary Fig. 2A). This highlights a need in the field for a methodology that can measure these marks separately from lower input samples, simultaneously in the same sample, and/or via direct readout.

Finally, because PD is an age-related disease and the epigenome has known age-related changes, it was surprising that including age as a covariate prevented proper fitting of the data, as demonstrated by a right-skewed *p*-value histogram, poor QQ plot, and genomic inflation value (lambda = 0.113) (Supplementary Fig. 2B,D). This observed genomic deflation may indicate overcorrection of the data. Using a model that is not well fitted to the data can lead to unreliable results and incorrect conclusions. Thus, we explored other covariate selections to identify a well-fitted model. Excluding age produced an appropriate *p*-value distribution and a QQ plot with moderate inflation (lambda = 1.22) (Supplementary Fig. 2A,C). This is consistent with both the known overestimation of inflation in EWAS studies and our inability to account for cell-type heterogeneity, as discussed above^90^. Attempts to correct for inflation and bias in these models did not improve the inflation (with age; lambda = 1.23) or made it worse (without age; lambda = 7.1). Overall, the model without age had a better relative fit to the data than the model with age. This unexpected fit could reflect the inability to account for cell-type variation as discussed above and/or that age has less of an effect on the interaction between 5mC and 5hmC than it does on overall DNA modification levels, as is measured in the majority of studies of aging and DNA modifications.

## Methods

### Human brain tissue selection

De-identified tissue samples from control (*n* = 50) and sPD (*n* = 50) human brain samples were obtained from archival human autopsy specimens provided by the Banner Sun Health Research Institute (BSHRI), using BSHRI’s approved institutional review board (IRB) protocols. Further details about the BSHRI’s brain samples and sample selection are available in a previous publication^91^. For each subject (*N* = 100), parietal cortex was obtained. This region develops pathology late in PD; in mid-stage PD it is expected to still have robust populations of neurons (unlike the substantia nigra, where neuron loss occurs early in disease), providing an avenue to investigate pre-pathological changes in gene regulation. We selected PD patients with mid-stage disease (Unified LB stage = II–IV), as defined by synuclein and Lewy pathology^92^. The cohort of control brains consisted of patients who died from non-neurologic causes and whose brains had no significant neurodegenerative disease pathology.

Selected PD cases did not meet diagnostic criteria for any other assessed neurological diagnosis included Alzheimer’s, dementia with Lewy bodies, vascular dementia, progressive supranuclear palsy, hippocampal sclerosis, dementia lacking distinctive histology, motor neuron disease, corticobasal degeneration, Pick’s disease, Huntington’s disease, multiple system atrophy, frontotemporal lobar dementia, or incidental Lewy body disease. A small subset of both PD and control subjects had cerebral amyloid angiopathy and mild cognitive impairment. The biggest difference between groups is that 31 of 50 PD cases, but no controls, had dementia without a defined dementing disorder or dementia lacking distinctive histology. Only 4 PD cases and 1 control case had tangles in the parietal cortex (0.5 tangle density). Parietal plaques were observed in both groups: 19 (PD), 24 (control).

### Magnetic-activated cell sorting

NeuN-positive (NeuN^+^) nuclei were enriched from 100 mg of flash frozen parietal cortex tissue using a two-stage magnetic-assisted cell sorting (MACS) method as previously described^19^. First, 100 mg of frozen tissue was briefly thawed on ice and homogenized in a 2 mL, 1.4 mm ceramic bead tube (Thermo Fisher Scientific, Cat. # 15-340-153) with 1 mL of Nuclear Extraction Buffer (NEB) for 10 s at 4 m/s. NEB consisted of 0.32 M sucrose, 0.01 M Tris-HCl pH 8.0, 0.005 M CaCl_2_, 0.003 M MgCl_2_, 0.0001 M EDTA, and 0.1% Triton X-100, up to a stock volume of 1 L using water. Immediately prior to use, 0.001 M DTT was added to NEB. Homogenized samples were loaded into a 13 mL ultracentrifuge tube (BeckmanCoulter, Cat. # 331372) with 4 mL of NEB. Using a glass pipette, 7 mL of sucrose solution was pipetted down the side of each sample tube to create a sucrose gradient. Sucrose solution consisted of 1.8 M sucrose, 0.01 M Tris-HCl pH 8.0, 0.003 M MgCl_2_, up to a stock volume of 1 L using water. Immediately prior to use, 0.001 M DTT was added to NEB. After addition of sucrose, samples were spun at 4 °C, 24,000 rpm in the Sorvall Wx+ Ultracentrifuge in a swing bucket rotor (TH-641). Once the centrifugation was complete, the supernatant and debris layer found at the concentration gradient were both removed with the use of a vacuum, while being careful not to disturb the pellet containing the nuclei at the bottom of the tube. Next, 1 mL of primary antibody (anti Neun 488—Millipore, Cat. # MAB377X) in MACS buffer was added to each nuclei pellet and placed on ice for 10 min. MACS buffer consisted of 0.5% Bovine Serum Albumin solution (Sigma-Aldrich, Cat. # A1595) in PBS pH 7.2 (Gibco, Cat. # 20012-027). Samples were then mechanically pipetted up and down 10−15 times to completely dissolve the nuclei pellet within the primary antibody-MACS buffer solution. This solution of nuclei was then transferred to a 2 mL tube and incubated for 60 min at 4 °C. After incubation, 40 µL of MACS Microbeads (anti-mouse IgG Microbeads - Miltenyi, Cat. # 130-048-401) were added to each sample. Samples were then inverted 4–5 times and incubated at 4 °C for 30 min. After incubation, nuclei were centrifuged at 300 x g for 10 min. Supernatant was then removed, and the nuclei were resuspended in 2 mL of MACS buffer and transferred to a MACS MS column (MS Separation columns – Miltenyi, Cat. # 130-042-201) that was pre-washed with MACS buffer and attached to the Miltyeni OctoMACS™ Separator. Positive selection of NeuN^+^ cells was then performed according to the standard MACS MS Columns protocol available from Miltenyi Biotec. After the first round of magnetic separation, NeuN^+^ nuclei were run through a separate, second MACS MS column to maximize cell type enrichment. To validate our methods, a subset of isolated nuclei was analyzed for flow cytometry on a CytoFlex S (Beckman Coulter), and data were analyzed using FlowJo V10, as reported in our previous study^19^. Percent positivity for each sample was defined as the percentage of events in the NeuN-A488+ gate, divided by the total number of events identified as Nuclei. The average proportion of neurons was estimated to be 83.8% across all samples.

### DNA extraction

DNA was isolated from enriched NeuN^+^ nuclei using the Qiagen QIAamp DNA MicroKit (Cat. # 56304) as previously described with some modifications to maximize yield^19^. Given that samples were already dissociated during nuclei isolation, the sample lysis and incubation steps of the QIAamp DNA Micro Kit protocol were removed. Instead, 20 µL of proteinase K were added directly to each MACS eluate. Samples were then vortexed for 15 s and incubated at room temperature for 15 min. In addition, the optional carrier RNA was added to Buffer AL, the incubation time after addition of 100% ethanol was increased to 10 min, the incubation time for the elution buffer was increased to 5 min, and the final elution step was repeated using 10 mM Tris-HCl pH 8.0.

### Oxidative bisulfite treatment and EPIC arrays

Intact genomic DNA yield was quantified by Qubit fluorometry (Life Technologies). Cleanup and preparation of DNA, and all steps for the EPIC bead chip protocol were performed as previously described per the manufacturer’s protocol, with additional steps for oxBS reactions^19^.

Bisulfite conversion was performed on 500 ng genomic DNA using the TrueMethyl Array kit (Cambridge Epigenetix). oxBS conversion was performed on 1 µg genomic DNA using the TrueMethyl Array kit (Cambridge Epigenetix). While the recommended amount of 500 ng DNA was sufficient for BS conversions, this was insufficient for oxBS, likely due to the additional oxidation step. These reactions were initially carried out on the same batch of DNA with 500 ng input for both reactions. However, with this input amount, all oxBS reactions failed to hybridize to the array. Thus, the oxBS reactions were run on a new batch of DNA isolations.

All conversion reactions were cleaned using SPRI-bead purification and eluted in Tris buffer. Following elution, BS- and oxBS-converted DNA was denatured and processed through the EPIC array protocol. The EPIC array contains ~850,000 probes that query DNA methylation at CpG sites across a variety of genomic features, including CpG islands, RefSeq genic regions, ENCODE open chromatin, ENCODE transcription factor binding sites, and FANTOM5 enhancer regions. To perform the assay, converted DNA was denatured with 0.4 N sodium hydroxide. Denatured DNA was then amplified, hybridized to the EPIC bead chip (v1.0 B5), and an extension reaction was performed using fluorophore-labeled nucleotides per the manufacturers protocol. Array BeadChips were scanned on the Illumina iScan platform.

### EPIC array data processing of oxBS data

IDAT files were imported into R and processed using an in-house bioinformatics pipeline that utilizes the following packages: *minfi* (version 1.48.0) for importing data, quality control, and dye bias correction, *ChAMP* (version 2.32.0) to perform singular value decomposition (SVD) to identify covariates for modeling, *posibatch* (version 1.0) for batch correction, and *ENmix* (version 1.38.01) to verify performance of control probes and perform a maximum likelihood estimate (MLE) of paired bisulfite and oxidative bisulfite, as previously described (Supplementary File 1)^19,20,22,93–97^. In this pipeline, minfi was used to import data, generate *β* values, and perform *ssNoob* background correction; *ENMix* was used to check the performance of control probes; *ChAMP* was used to assess data before and after normalization. After normalization, data were split by assay (BS and oxBS) prior to SVD analysis (*ChAMP)* and batch correction *(posibatch)* (Supplementary Fig. 1).

After QC, eleven female samples and six male samples were removed due to a high level (>10%) of failed probes, leaving 57 male (28 PD, 29 control) and 26 female (13 PD, 13 control) samples. One additional male sample was excluded because it was excluded in our previous BS-only analysis, leaving 56 male samples (27 PD, 29 control) (47). Failed probes (53,305) were removed from remaining samples when detection *p*-value was >0.01 in >5% of samples. Cross-reactive probes and probes containing SNPs (93,527) were masked based on previous identification^98^.

We continued with male samples for full analysis only due to the small sample size of the remaining female samples. For female samples, we restricted analysis to the male iDMCs.Data for the included samples are summarized in Table 6 and all included metadata is in Supplementary File 2. This study has a sample size smaller than recent recommendations published by Mansell et al. for BS-only-based studies^99^. Using a cohort experimentally controlled for multiple variables and sorting for specific cell types as we did here, may help to reduce variability. However, this sample size highlights a challenge in the field for brain-specific EWAS, where sample sizes are often limited by brain bank availability. Most existing power estimators for EPIC array data do not include brain-specific methylation data; power in EWAS studies has been shown to be highly tissue-specific for case-control studies^100^. While newer publications provide needed information for brain-specific EWAS, these are based on BS-based analysis only. Our study is based on the post-MLE processed 5mC and 5hmC *β* values from the enriched population of neuronal nuclei isolated by MACS and confirmed by flow cytometry. Overall, there is a lack of existing data on which to base power analysis for this type of data. Despite these limitations, our BS-only study remains the largest neuron-enriched PD brain EWAS to our knowledge, and the current study is the only PD study examining paired BS/oxBS data^19^. Future studies should include larger cohorts or be combined with publicly available data. In addition, this highlights the need for additional studies and meta-analysis of multiple studies, as has been conducted to combine smaller EWAS studies for Alzheimer’s disease^101^.

**Table 6:** Cohort characteristics of included samples

Male (*n* = 56)			Female (*n* = 26)	
Variables	Mean ± SD or *N* (%)	Range	Mean ± SD or *N* (%)	Range
*Disease Status*				
Control	29 (51.8%)		13 (50%)	
Parkinson’s disease	27 (48.2%)		13 (50%)	
*Age at Death*				
Control	79.3 ± 9.1	53-93	83.7 ± 13.7	52-95
Parkinson’s disease	79.4 ± 7.1	64-91	78.1 ± 5.3	70-87
*PMI*				
Control	3.25 ± 0.81	2.16-5.5	3.2 ± 0.8	2.25-5
Parkinson’s disease	3.27 ± 0.83	1.83-4.92	3.2 ± 0.8	1.75-4.4
*Race*				
White	55 (98.2%)		13 (50%)	

Data includes disease status, age at death in years, postmortem interval (PMI) in hours, and race of samples remaining after QC. Seven male samples and eleven female samples were removed during quality control and pre-processing, leaving 56 male and 26 female samples.

5hmC *β* values (β_hmC_) were estimated by pairing oxBS *β* values with BS *β* values using the maximum likelihood estimate function (oxBS.MLE*)* from the *ENmix* package, which returns true methylation *β* values (β_mC_) from oxBS reactions and estimates β_hmC_. Density plots of raw BS and oxBS *β* values, as well as MLE-corrected β_mC_ and β_hmC_ values, are shown in Fig. 1A, B.

After MLE, probes with mean β_mC_ or β_hmC_ < 0.01 across all samples were removed due to increased variability and decreased interpretability of *β* values at such low levels, as well as to remove the issue of zero inflation for 5hmC *β* values. After all QC steps, there were 587,065 probes where β_mC_ > 0.01 and β_hmC_ > 0.01 (714,427 probes had β_mC_ > 0.01, and 587,091 probes had β_hmC_ > 0.01. Of these, 26 probes had β_hmC_ data only, and 127,362 had β_mC_ data only.

#### Differential testing for differentially methylated cytosines

The *gamlss* (Generalized Additive Models for Location, Scale, and Shape) R package (version 5.4-22) was used to test for interaction differentially methylated cytosines (iDMCs) as a site where there is a shift in the balance between 5mc and 5hmc, as previously described (Supplementary File 3)^20,102^. Briefly, the mixed effects model treats 5mc and 5hmc as “repeated” measures of a single outcome variable (DNA modification), and a random effect for ID accounts for the correlation between 5mc and 5hmc at a CpG site. Meanwhile, a “DNA modification*Experimental Condition” interaction term is used to determine if 5mc and 5hmc differ in their response to the experimental condition. PMI was included as a covariate because it was identified as a significant principal component by SVD analysis in *ChAMP* (Supplementary Fig. 1). Sex was not included because only male samples were included. Glial cell content was not used as a covariate in this study, as it was in our previous study, since oxBS reactions were run on a separate batch of nuclei and DNA isolations due to the failure of the oxBS reactions. This is a technical variable we are unable to control for in this study, as datasets for glial cell estimates are based on BS data only and may contribute to the observed inflation (Supplementary Fig. 1). Age was not included because including age in the model resulted in poor performance of the model (Supplementary Fig. 2). Including age as a covariate resulted in poor model fit consistent with overcorrection. Removing age as a co-variate produced the expected *p*-value histogram and slight genomic inflation This inflation is consistent with (1) the known overestimation of inflation in EWAS studies and (2) unaccounted for variation from not including cell-type heterogeneity estimates as noted above^90^. We attempted to control for inflation and bias by using the *bacon* package^90^. However, in the model without age, this did not improve the inflation, and in the model with age, it worsened the inflation.

An FDR < 0.05 was used as the cutoff for significance, and annotation of significant differential probes was performed using the Illumina EPIC array manifests. For the restricted analysis in female samples, *p*-value < 0.05 was used. QQ plots generated using the R package *QCEWAS* (version 1.2.3) and *ggplot2*, respectively, with lambda = 1.22 for interaction modeling. A lambda > 1 suggests that observed *p*-values are more significant than expected by chance, and this data may have some unaccounted technical variation. When age is included as a covariate, lambda = 0.113 (Supplementary Fig. 2).

#### Annotation of interaction DMCs

Gene IDs corresponding to each iDMC were extracted from the EPIC array manifest provided by Illumina (v1.0 B5). Annotation of universal chromatin states was also performed using the *annotatr* R package (version 1.28.0) and adding custom full stack ChromHMM chromatin states for *hg38* to the annotation cache^103^. For specific candidate loci, brain-specific imputed ChromHMM annotations for cingulate gyrus (E069) and SN (E074) were used; ChromHMM annotations are not available for parietal cortex^103^. Enrichment of iDMCs within genomic features and chromHMM annotations was performed with the EPIC array as background. Since this study utilized neuronally enriched nuclei, neuronal expression of iDMC-containing genes of interest was verified using the Allen Brain Cell Atlas^67^. Specific loci were also compared to a database of imprint control regions (ICR)^44^. UpSet plots comparing these results with previous EWAS studies were generated using the *UpSetR* package (version 1.4.0)^104^.

#### Predicting enhancer targets

iDMCs annotated to weak enhancers, active enhancers, and transcribed enhancers based on ChromHMM chromatin state annotations were input to GREAT to predict target genes of these iDMC-containing enhancers^105^. The basal plus extension method for association of genes was used, with curated regulatory domains included.

#### Gene ontology pathway enrichment

Gene ontology (GO) term enrichment testing and pathway analysis were performed using a combined list of unique iDMC-containing genes and targets of iDMC-containing enhancer regions using the ClueGO application in Cytoscape (version 3.10.1)^106,107^. Because we included the genes identified as targets of iDMC-containing enhancers that did not necessarily contain iDMCs, we did not use a GO method that accounts for biased array probe coverage. “Groups” was selected as the visual style, and the GO Biological Process (GOBP) term and the GO Cellular Component term were selected. Network specificity was set to “Medium”, with the GO Tree Interval minimum set at 3 and maximum at 8. Only terms with at least 3 genes and a Bonferroni-corrected *p*-value < 0.05 were included in pathway visualizations. The connectivity score (Kappa) was set at 0.4, and default GO Term Grouping settings were used in all analyses.

#### Protein-protein interaction networks

The same list of genes was also used for protein-protein interaction network analysis with STRING (version 12.0) using a minimum required interaction score = 0.7 and all other default parameters^108^. The minimum required confidence level was high > = 0.7.

### Ethics approval and consent to participate

De-identified tissue samples from control (*n* = 50) and Parkinson’s disease (*n* = 50) human brain samples were obtained from archival human autopsy specimens provided by the Banner Sun Health Research Institute (BSHRI), using BSHRI’s approved institutional review board (IRB) protocols.

## Supplementary information

Supplementary Data

Supplementary Data1

Supplementary Data2

Supplementary Data3

Supplementary Data4

Supplementary Data5

Supplementary Data6

Supplementary Data7

Supplementary Data8

Supplementary Data9

Supplementary Data10

Supplementary Data11

Supplementary Data12

## Data Availability

This study was preregistered with Open Science Framework: https://osf.io/z4vbw. Raw and processed data are available in GEO (GSE267937): https://www.ncbi.nlm.nih.gov/geo/query/acc.cgi?acc=GSE267937 All supplementary material, including additional figures, tables of results, and code used for analyses, are available as supplementary files.
