# Supplementary material for: Parkinson’s disease-associated alterations in DNA methylation and hydroxymethylation in human brain
Source: NPJ Parkinsons Dis. 2025 Dec 22;11:363. doi: 10.1038/s41531-025-01209-3 (PMC12749594; doi:10.1038/s41531-025-01209-3)
Supplement: Supplementary file 1 — Supplementary Data [file 41531_2025_1209_MOESM1_ESM.pdf]

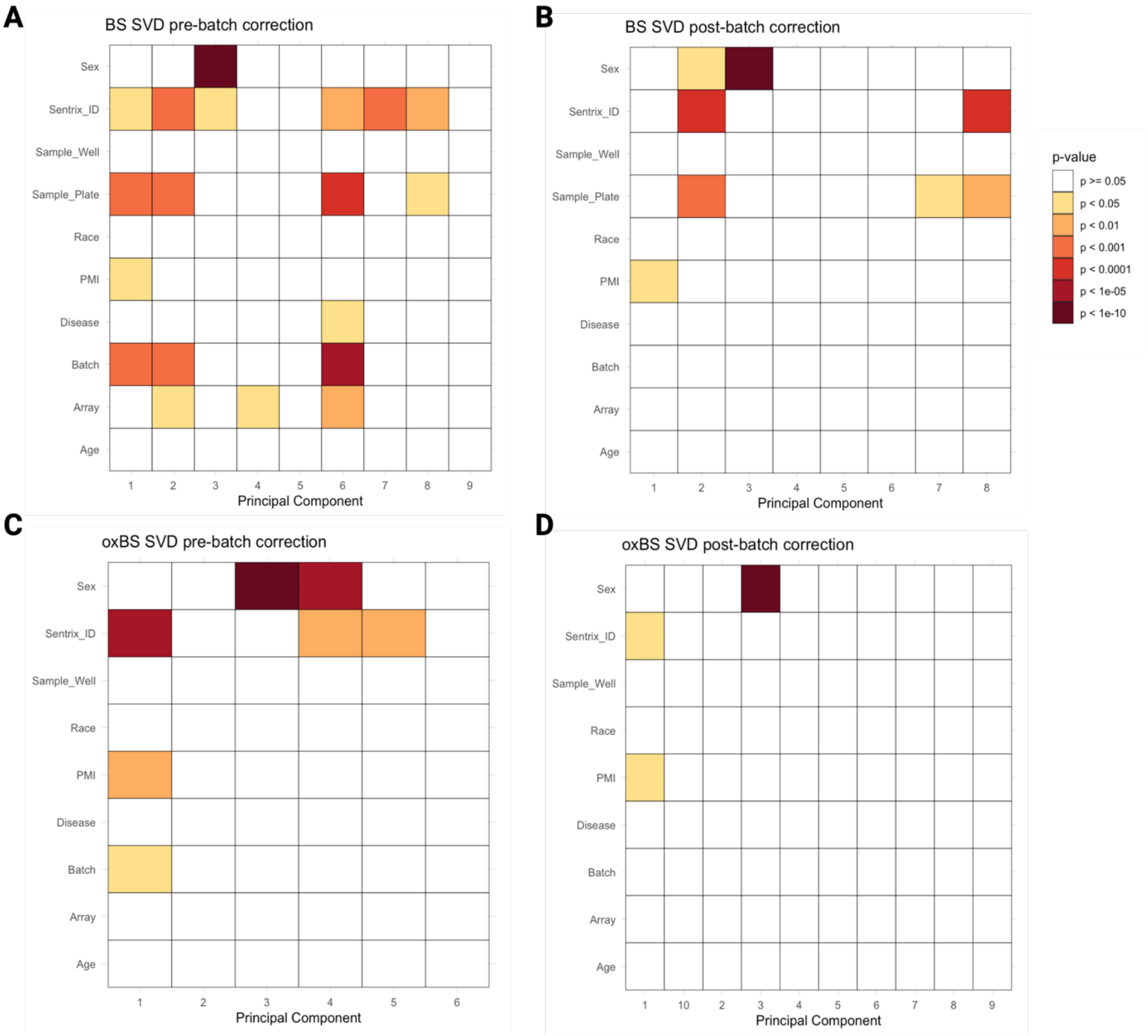

Supplementary Figure 1: Singular value decomposition plots pre- and post-batch correction. *ChAMP* was used to identify batch effects in BS and oxBS data separately. SVD plots show which variables contribute significantly to each principal component. (A) BS data shown pre-batch correction and (B) post-batch correction with *posibatches* (C) oxBS data shown pre-batch correction and (D) post-batch correction. Comparison of SVD blots pre- and post-batch correction indicate that batch correction performed as expected. Colors indicate significant p-values ($p < 0.05$) as shown in the legend. Non-significant p-values ($p \geqq 0.05$) are shown as white.

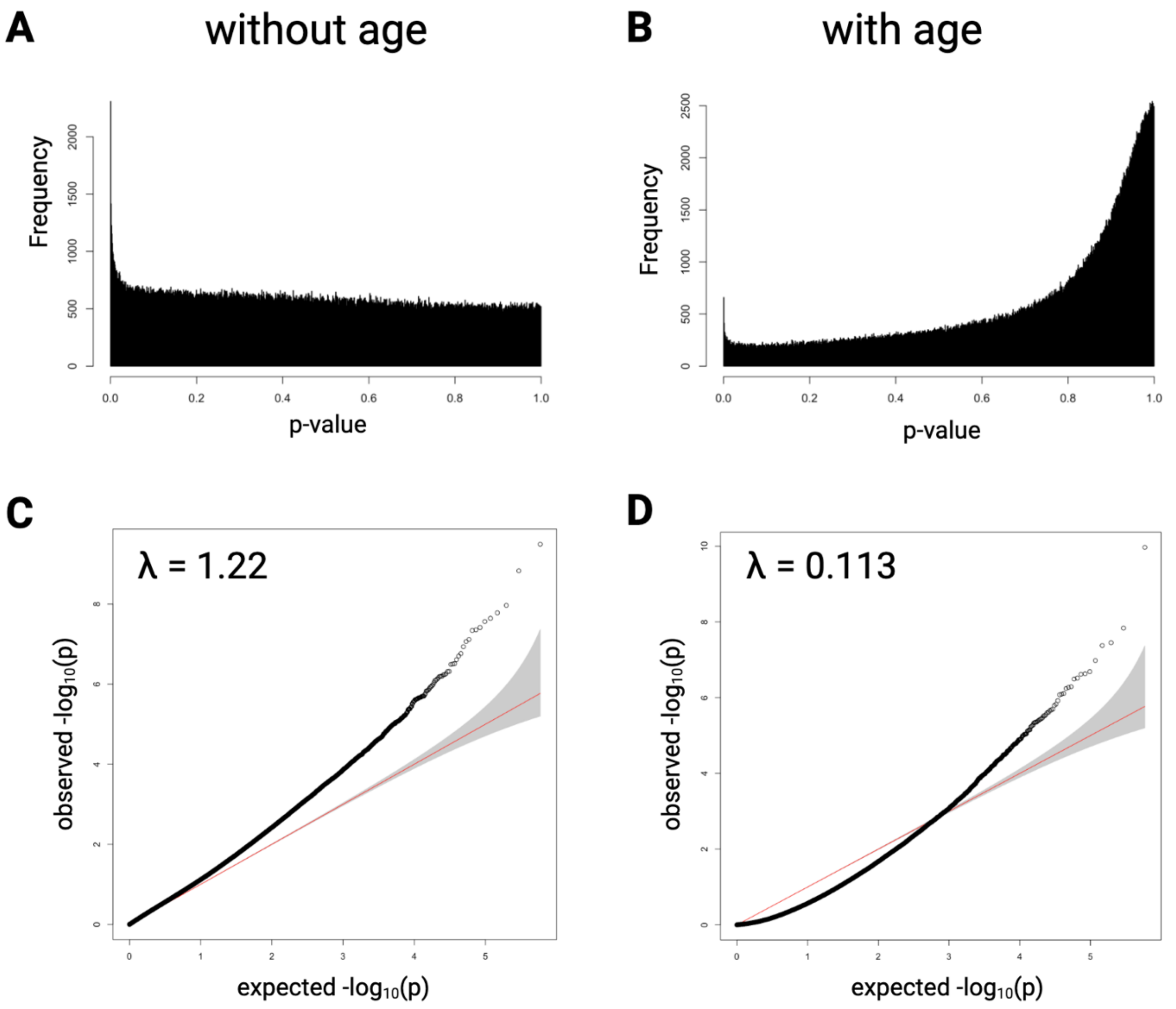

Supplementary Figure 2: Histograms of p-values and QQ plots. P-value histograms (A,B) and QQ plots (C,D) were generated for the interaction modeling with (B,D) and without (A,C) age as a covariate. The p-value histogram for modeling without age as a covariate (A) shows an appropriate distribution with a peak near 0, indicating that the null hypothesis is false for a subset of tests, while the p-value histogram with age as a covariate (B) is skewed to the right, indicating that this model does not fit the data well. (C) QQ plots show lambda = 1.22 for modeling without age, indicating moderate inflation. This suggests that observed p-values are more significant than expected by chance, and there may be unaccounted-for variation. As we were unable to control for cell-type heterogeneity in this study, this is consistent with unaccounted-for variation. This observed inflation is also consistent with the known overestimation of genomic inflation in EWAS (van Iterson 2017, PMID: 28129774). Running *bacon* to control for inflation and bias, also produced an appropriate p-value histogram but did not reduce the inflation (lambda = 1.23). (D) When age is included as a covariate, lambda = 0.113, indicating severe deflation and potential

overcorrection of the model, consistent with the p-value histogram showing poor fit. For this model, running bacon produced an appropriate p-value distribution, but showed extreme genomic inflation with a lambda of >7.1.

Supplementary data legends and column definitions

Supplementary_Data_1.Rmd: This Rmd file contains code for importing paired BS/oxBS data from the Illumina MethylationEPIC array, performing QC and pre-processing steps, and producing MLE beta value estimates.

Supplementary_Data_2.csv: This file contains metadata used in the analysis and has the following columns:

Sample_ID: unique identifier for each subject
subject: unique coded identifier for each subject
Title: assay title including nuclei fraction, assay, and subject
Index: unique identifier for each reaction
Sample_Name: sample title including Sample ID, nuclei fractions and assay
Assay: BS assays is coded as 1, oxBS assay is coded as 2
Assay_1: oxBS or BS
Disease: PD or control
Disease_coded: control is coded as 1, PD is coded as 2
Race: 1 is white, 99 is unidentified
Gender: 1 is male, 2 is female
Age: age in years at time of death
PMI: postmortem interval in hours
All other columns are standard EPIC Array Sample Sheet columns.

Supplementary_Data_3.Rmd: This Rmd file contains code that tests for differentially methylated and hydroxymethylated CpGs using the paired gamlss model with interaction term. This file uses the output generated by the code in Supplementary_Data_1_EPIC_QC_MLE.Rmd.

Supplementary_Data_4.csv: This file contains the output of the differential analysis code in Supplementary_Data_3_.Rmd as csv. It contains annotated significant probes (FDR <0.05) and has the following columns:

Probe: EPIC array probe ID
Estimate: interaction term (β coefficient)
Pr...t..:

fdr_m: adjusted p-values using the Benjamini-Hochberg FDR adjustment method
Std..Error: standard error
t.value: t statistic for estimated interaction term

Selected columns from the EPIC manifest (v1.0 B5) containing hg19 genomic locations, genic annotations, if these occur in experimentally determined DMRs, and the hg38 genomic coordinates:

CHR: chromosome containing the CpG (hg19)
MAPINFO: chromosomal coordinates of the CpG (hg19)
UCSC_RefGene_Name: NCBI RefSeq Gene Name: Target gene names from the RefSeq database. Multiple listings of the same gene name indicate splice variants
UCSC_RefGene_Accession: NCBI RefSeq Transcript ID: The RefSeq accession numbers of the target transcripts. Accession numbers are in the same order as the target gene transcripts
UCSC_RefGene_Group: NCBI RefSeq Gene region: 5UTR=5' untranslated region between the TSS and ATG start site, 3UTR=3' untranslated region between stop codon and poly A signal, exon_#, TSS200=1-200 bp 5' the TSS, TS1500=200-1500 bp 5' of the TSS. Intronic regions included
DMR: Differentially methylated regions (experimentally determined). DMR = Differentially Methylated Region. CDMR = Cancer-specific Differentially Methylated Region. RDMR = Reprogramming-specific Differentially Methylated Region.
CHR_hg38: chromosome containing CpG (hg38)
Start_hg38: start position of chromatin state feature (hg38)
End_hg38: end position of chromatin state feature (hg38)
Strand_hg38: strand info of chromatic state feature (hg38)

Selected columns from human full-stack chromatin state annotation in hg38 assembly containing genomic location and annotation of each genomic features:

Seqnames: chromosome number of probe
start: start position of probe
end: end position probe
width: length of segment in base pairs
strand: strand info of probe
annot.seqnames: chromosome number of annotated feature
annot.start: start position of annotated feature
annot.end: end position of annotated feature

annot.width: length of annotated feature in base pairs
annot.strand: DNA strand of annotated feature
annot.id: unique identifier for annotated feature
annot.tx_id: transcript ID
annot.gene_id: gene ID
annot.symbol: human readable gene symbol
annot.type: type of genomic feature

Supplementary_Data_5.csv: contains iDMCs identified in male subjects that have p-value < 0.05 in samples from female subjects.

probe: EPIC array probe ID
beta.coefficient: estimated interaction term in male samples
SE: standard error in male samples
p.value: p-value in male samples
FDR: adjusted p-values using the Benjamini-Hochberg FDR adjustment method
beta.coefficient.f: estimated interaction term in female samples
SE.f: standard error in female samples
p.value.f: p-value in female samples
CHR: chromosome containing the CpG (hg19)
MAPINFO: chromosomal coordinates of the CpG (hg19)
UCSC_RefGene_Name: NCBI RefSeq Gene Name: Target gene names from the RefSeq database. Multiple listings of the same gene name indicate splice variants
UCSC_RefGene_Group: NCBI RefSeq Gene region: 5UTR=5' untranslated region between the TSS and ATG start site, 3UTR=3' untranslated region between stop codon and poly A signal, exon_#, TSS200=1-200 bp 5' the TSS, TS1500=200-1500 bp 5' of the TSS. Intronic regions included

Supplementary_Data_6.txt: List of 83 genes containing iDMCs from male subjects

Supplementary_Data_7.txt: List of 67 genes identified as targets of iDMC-containing enhancers identified using GREAT.

Supplementary_Data_8.txt: Combined unique list of 134 iDMC containing genes and GREAT genes used for subsequent analysis.

Supplementary_Data_9.csv: This file contains the annotated DMC output for the BS only analysis generated by Supplementary_Data_11_BSonly_DiffMeth.Rmd.

Name: EPIC array probe ID

model.5mC.Estimate: average 5mC beta value
model.5mC.Pr...t..: p-value
fdr.5mC: adjusted p-values using the Benjamini-Hochberg FDR adjustment method
model.5mC.Std..Error: standard error
model.5mC.t.value: t statistic
qvalue: FDR calculated with qvalue package
sex: male or female subjects
All EPIC Array columns from manifest (v1.0 B5)

Supplementary_Data_10: This file contains the annotated DMR output from DMRcate of the code in Supplementary_Data_11_BSonly_DiffMeth.Rmd

seqnames: EPIC array probe ID
start: start position of DMR
end: end position of DMR
meandiff: average difference in beta values between groups
min_smoothed_fdr: minimum smoothed FDR for multiple testing correction
width: length of DMR in base pairs
strand: strand of DMR
no.cpgs: number of CpG sites in DMR
Stouffer: p-value from Stouffer's method
HMFDR: harmonic mean of FDRs
Fisher: p -value generated by Fisher's test
maxdiff: maximum difference in beta values between groups
overlapping.genes: gene symbol of any gene that overlaps DMR
sex: male or female subjects

Supplementary_Data_11.Rmd: This file contains the code for re-analyzing previously processed BS-only beta values that returns differentially modified cytosines and differentially modified regions.

Supplementary_Data_12.xlsx: This file contains overlapping genes between this study and other recent brain-specific EWAS studies for PD.
